# Self‐supported bimetallic array superstructures for high‐performance coupling electrosynthesis of formate and adipate

**DOI:** 10.1002/EXP.20230043

**Published:** 2023-12-21

**Authors:** Li Liu, Yingchun He, Qing Li, Changsheng Cao, Minghong Huang, Dong‐Dong Ma, Xin‐Tao Wu, Qi‐Long Zhu

**Affiliations:** ^1^ State Key Laboratory of Structural Chemistry, Fujian Institute of Research on the Structure of Matter Chinese Academy of Sciences Fuzhou China; ^2^ Fujian Science & Technology Innovation Laboratory for Optoelectronic Information of China Fuzhou China; ^3^ University of Chinese Academy of Sciences Beijing China; ^4^ School of Civil and Environmental Engineering University of Technology Sydney Ultimo New South Wales Australia

**Keywords:** coupling electrosynthesis, electrocatalysis, superstructures

## Abstract

The coupling electrosynthesis involving CO_2_ upgrade conversion is of great significance for the sustainable development of the environment and energy but is challenging. Herein, we exquisitely constructed the self‐supported bimetallic array superstructures from the Cu(OH)_2_ array architecture precursor, which can enable high‐performance coupling electrosynthesis of formate and adipate at the anode and the cathode, respectively. Concretely, the faradaic efficiencies (FEs) of CO_2_‐to‐formate and cyclohexanone‐to‐adipate conversion simultaneously exceed 90% at both electrodes with excellent stabilities. Such high‐performance coupling electrosynthesis is highly correlated with the porous nanosheet array superstructure of CuBi alloy as the cathode and the nanosheet‐on‐nanowire array superstructure of CuNi hydroxide as the anode. Moreover, compared to the conventional electrolysis process, the cell voltage is substantially reduced while maintaining the electrocatalytic performance for coupling electrosynthesis in the two‐electrode electrolyzer with the maximal FE_formate_ and FE_adipate_ up to 94.2% and 93.1%, respectively. The experimental results further demonstrate that the bimetal composition modulates the local electronic structures, promoting the reactions toward the target products. Prospectively, our work proposes an instructive strategy for constructing adaptive self‐supported superstructures to achieve efficient coupling electrosynthesis.

## Graphical abstract

The self‐supported bimetallic array superstructures were constructed for high‐performance coupling electrosynthesis of formate and adipate at the anode and cathode, respectively. Thanks to the unique nanostructure features and the promotion effect of adaptive reactions, the resulting electrocatalysts delivered remarkable performance for both CO2‐to‐formate and cyclohexanone‐to‐adipate conversion with high Faradaic efficiencies and current densities.

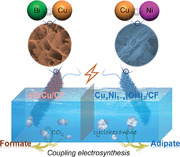

## 1 INTRODUCTION

Different from other energy and environment‐related reactions, electrochemical carbon dioxide reduction to produce valuable products can realize efficient utilization of intermittent renewable energy, which is an effective way to reduce the concentration of CO_2_ in the atmosphere and promote a sustainable closed carbon cycle.^[^ ^1^ ^]^ It holds great significance in mitigating global warming and reducing the depletion of traditional energy resources.^[^ ^2^ ^]^ Specifically, a typical CO_2_ electroconversion system includes a cathodic CO_2_ reduction reaction (CO_2_RR) and the anodic oxygen evolution reaction (OER). However, the slow OER necessitates a high onset potential, significantly limiting the rate of electrochemical CO_2_RR and resulting in substantial energy losses.^[^ ^3^ ^]^ As a result, to fully harness the electrical energy, a more kinetically favorable oxidation reaction should be used to replace the OER, which is also expected to produce value‐added products.^[^ ^4^ ^]^

**SCHEME 1:**
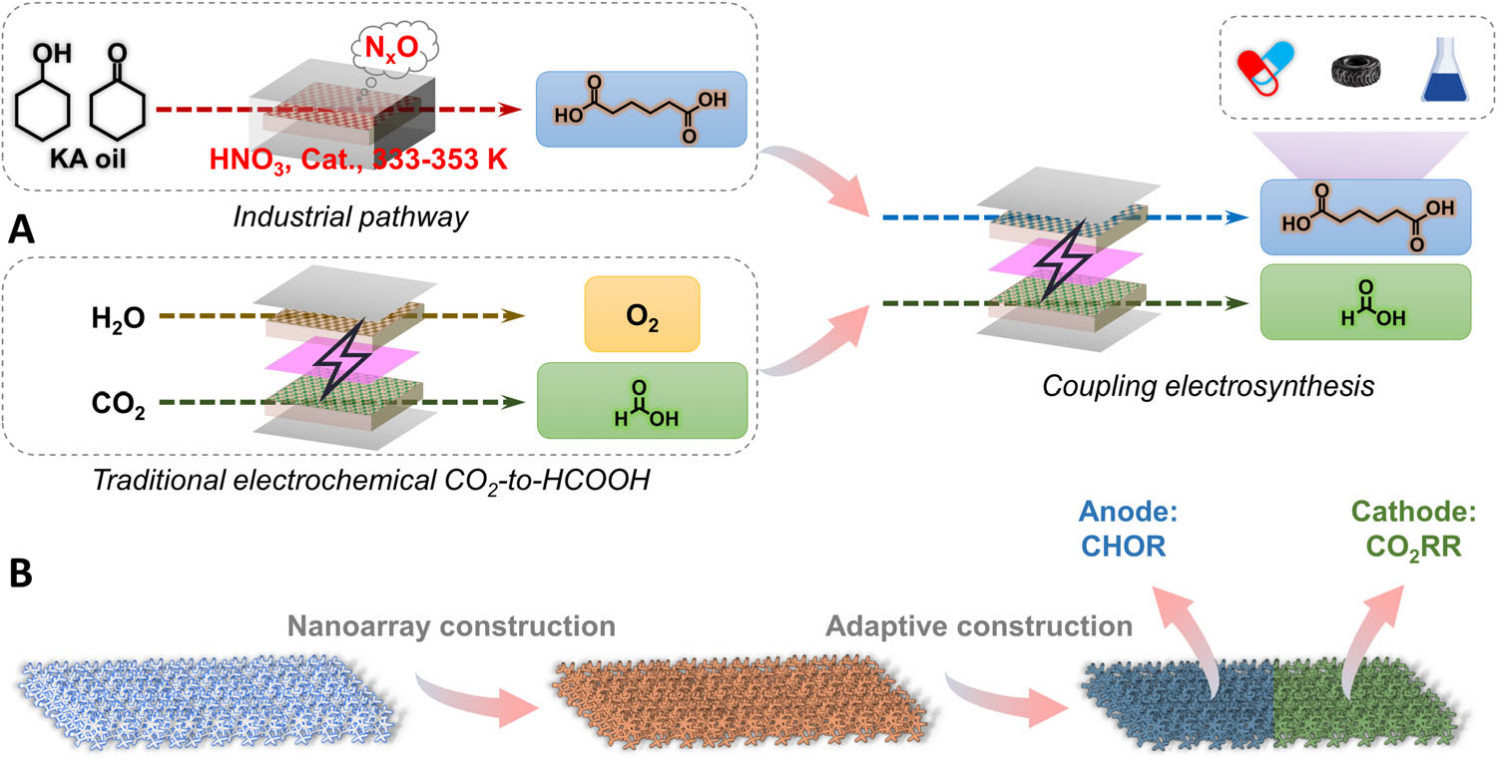
A, Schematic illustration of upgrading the traditional (electro)synthesis to coupling electrosynthesis of formate and adipate, and B, fabricated illustration of electrocatalysts for coupling electrosynthesis.

Adipic acid is a pivotal molecule in the polymer industry, possessing a broad range of applications and experiencing high demand.^[^ ^5^ ^]^ However, the current method for industrial adipic acid preparation relies on the thermo‐catalytic oxidation of KA oil (a combination of cyclohexanone and cyclohexanol) under harsh conditions involving the use of highly corrosive nitric acid as an oxidant. This process causes significant energy consumption and results in hazardous gas emissions.^[^ ^6^ ^]^ Therefore, it is highly desirable to seek a more environmentally friendly and sustainable adipic acid synthesis process.^[^ ^7^ ^]^ Electrocatalytic cyclohexanone oxidation reaction (CHOR) driven by electrical energy offers an excellent opportunity for adipic acid production toward a more sustainable chemical industry.^[^ ^8^ ^]^ Particularly and prospectively, coupling electrochemical CO_2_RR with CHOR can not only reduce the energy input of the electrolysis system but also realize the green electrochemical synthesis of adipic acid (Scheme 1A). However, the key to achieving this coupling electrosynthesis lies in developing highly active and stable electrocatalysts. Notably, among many materials, the self‐supported electrocatalytic materials with bimetallic configuration can realize the accurate integration of morphology engineering and electronic structure engineering,^[^ ^9^ ^]^ which are expected to propel efficient coupling of cathodic CO_2_RR and anodic CHOR for integrated industrial production; however, there is a lack of in‐depth systematic exploration.

Herein, according to the characteristics of cathodic CO_2_RR and anodic CHOR, we hired Cu(OH)_2_ nanowire arrays grown on copper foam (CF) as the three‐dimensional self‐supported substrate, and Bi (one of the most promising active elements for electrochemical CO_2_‐to‐formate conversion^[^ ^10^ ^]^) and Ni species were, respectively, introduced to construct suitable electrocatalysts (namely, eBiCu/CF and Cu_x_Ni_1−x_(OH)_2_/CF) (Scheme 1B). Expectedly, in situ electrochemically reconstructed eBiCu/CF consists of porous and ultrathin alloy nanosheets with an open network structure, which achieves more than 90% faradaic efficiency for formate (FE_formate_) in a wide potential range, far superior to the counterpart electrocatalysts, and Cu_x_Ni_1−x_(OH)_2_/CF features a nanosheet‐on‐nanowire array structure with ultrathin hydroxide nanosheets interconnected and welded on the surface of nanowires, which exhibits excellent electrocatalytic performance for CHOR with faradaic efficiency of adipate (FE_adipate_) up to 95.7%. In particular, the coupling electrosynthesis system of CO_2_RR//CHOR was further assembled to simultaneously achieve >90% FE_formate_ and FE_adipate_, showing great energy‐saving and value‐added benefits. It is demonstrated that the unique bimetallic cooperative electronic structures can promote the electrocatalytic efficiencies of target products.

## 2 RESULTS AND DISCUSSION

### 2.1 Characterizations and discussion of eBiCu/CF

**FIGURE 1:**
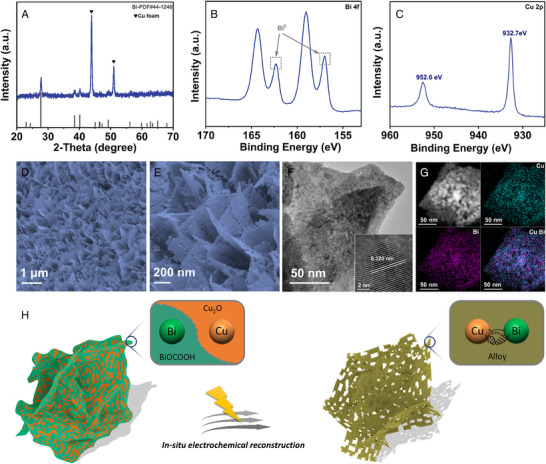
Characterization of eBiCu/CF: A, X‐ray diffraction (XRD) pattern, x‐ray photoelectron spectroscopy (XPS) spectra for B, Bi 4f and C, Cu 2p, D,E, SEM images, F, transmission electron microscopy (TEM) and G, energy‐dispersive x‐ray (EDX) elemental mapping images, and H, schematic illustration of in situ electrochemical reconstruction.

The eBiCu/CF electrocatalytic material was first prepared by in situ electrochemical reconstruction of the self‐supported Bi‐O‐Cu/CF electrode that was derived from vertically aligned Cu(OH)_2_ nanowire arrays on the CF (Figures S1–S3). After in situ electrochemical reconstruction, eBiCu/CF was immediately characterized by x‐ray diffraction (XRD). As shown in Figure S3A and Figure 1A, XRD patterns prove the successful electroconversion of Bi‐O‐Cu/CF to eBiCu/CF. Concretely, the diffraction peaks of BiOCOOH and Cu_2_O in Bi‐O‐Cu/CF disappear, and three distinguishable peaks at 27.52°, 38.40°, and 40.04° appear in eBiCu/CF, matching the hexagonal Bi (JCPDS No.44‐1246). This indicates that Cu‐doped ultrathin Bi nanosheets may be formed in the resulting eBiCu/CF. Furthermore, x‐ray photoelectron spectroscopy (XPS) studies also confirmed the phase transition and bimetallic interaction (Figure 1B,C and Figure S3B,C). It can be seen that in the C 1s spectrum, the signal peak of HCOO^–^ in BiOCOOH disappears after in situ electrochemical conversion. At the same time, through the analysis of the Bi 4f spectrum, we can find the attendance of the Bi^0^ peaks, and the Bi^3+^ peaks also undergo significant displacement in eBiCu/CF. It is worth noting that the strong Bi^3+^ peaks in eBiCu/CF are attributed to the inevitable surface oxidation of the sample during characterization. In addition, the Bi^0^ peaks in eBiCu/CF appear at 162.4 and 157.0 eV, which are lower than pure Bi (162.7 and 157.3 eV), indicating an increase in the electron density of Bi atoms in eBiCu/CF.^[^ ^11^ ^]^ Meanwhile, the Cu^0^ peaks in eBiCu/CF are located at 952.6 and 932.7 eV, which is slightly higher than those of pure Cu.^[^ ^12^ ^]^ These results indicate the existence of electron transfer from Cu to Bi in eBiCu/CF, resulting in electron‐rich Bi and consequently its superior activity and selectivity for CO_2_RR.^[^ ^13^ ^]^

Second, after in situ electrochemical transformation, the obtained eBiCu/CF catalyst maintains the nanosheet array morphology with a three‐dimensional porous network structure (Figure 1D,E), which is beneficial for CO_2_ diffusion and rapid electrolyte penetration, potentially achieving excellent electrochemical CO_2_RR performance. Meanwhile, the transmission electron microscopy (TEM) image in Figure 1F indicates that many small pores are implanted on the surface of eBiCu nanosheets. According to the literature, the detachment of non‐metal atoms leads to the size contraction of the catalyst during electrochemical reconversion, thus producing abundant edge sites and porous morphology.^[^ ^10^, ^14^ ^]^ The lattice fringe in the high‐resolution TEM (HRTEM) image with a distance of 0.320 nm corresponds to the (012) crystal plane of Bi, thereby demonstrating the conversion of BiOCOOH to Bi (Figure 1F, inset). In addition, the energy‐dispersive x‐ray (EDX) image shows that Cu and Bi are uniformly distributed throughout the nanosheets, and the ratio of Cu and Bi is consistent with that in Bi‐O‐Cu/CF (Figure 1G and Figure S4). In a word, we have verified the successful preparation of eBiCu/CF through the above characterization, and its rough and porous ultrathin nanosheet array superstructure, as well as abundant edge sites (Figure 1H), are expected to achieve excellent electrocatalytic activity in subsequent CO_2_RR.^[^ ^14^, ^15^ ^]^

### 2.2 Electrochemical performance of eBiCu/CF for CO_2_RR

The electrocatalytic CO_2_RR performance of eBiCu/CF was evaluated in the CO_2_‐saturated 0.5 M KHCO_3_ solution in an H‐type electrolytic cell. We first compared the electrochemical CO_2_RR performance of CF, Cu(OH)_2_/CF, and eBiCu/CF electrodes. Figure S5 shows the linear sweep voltammogram (LSV) curves in the CO_2_‐ and Ar‐saturated 0.5 M KHCO_3_ solution. Compared with the Ar‐saturated electrolyte, the current density significantly increased in the CO_2_‐saturated electrolyte, indicating that electrochemical CO_2_RR occurred on the eBiCu/CF electrode. To study the product distribution of electrochemical CO_2_RR, electrolysis was conducted at various potentials between −0.58 and −1.28 V for 1 h, and the gas and liquid products were quantitatively analyzed by gas chromatography (GC) and nuclear magnetic resonance (NMR) spectroscopy, respectively (Figure S6). The electrocatalytic results of eBiCu/CF indicate that formate is the only liquid product, with small amounts of H_2_, CO, and CH_4_ gas products, while CF and Cu(OH)_2_/CF mainly produce H_2_ (Figure S7). Further analysis shows that the *j*_formate_ of eBiCu/CF is much larger than those of CF and Cu(OH)_2_/CF (Figure S8). It is found that the high selectivity of CO_2_‐to‐formate conversion strongly depends on the introduction of Bi on the electrode.

**FIGURE 2:**
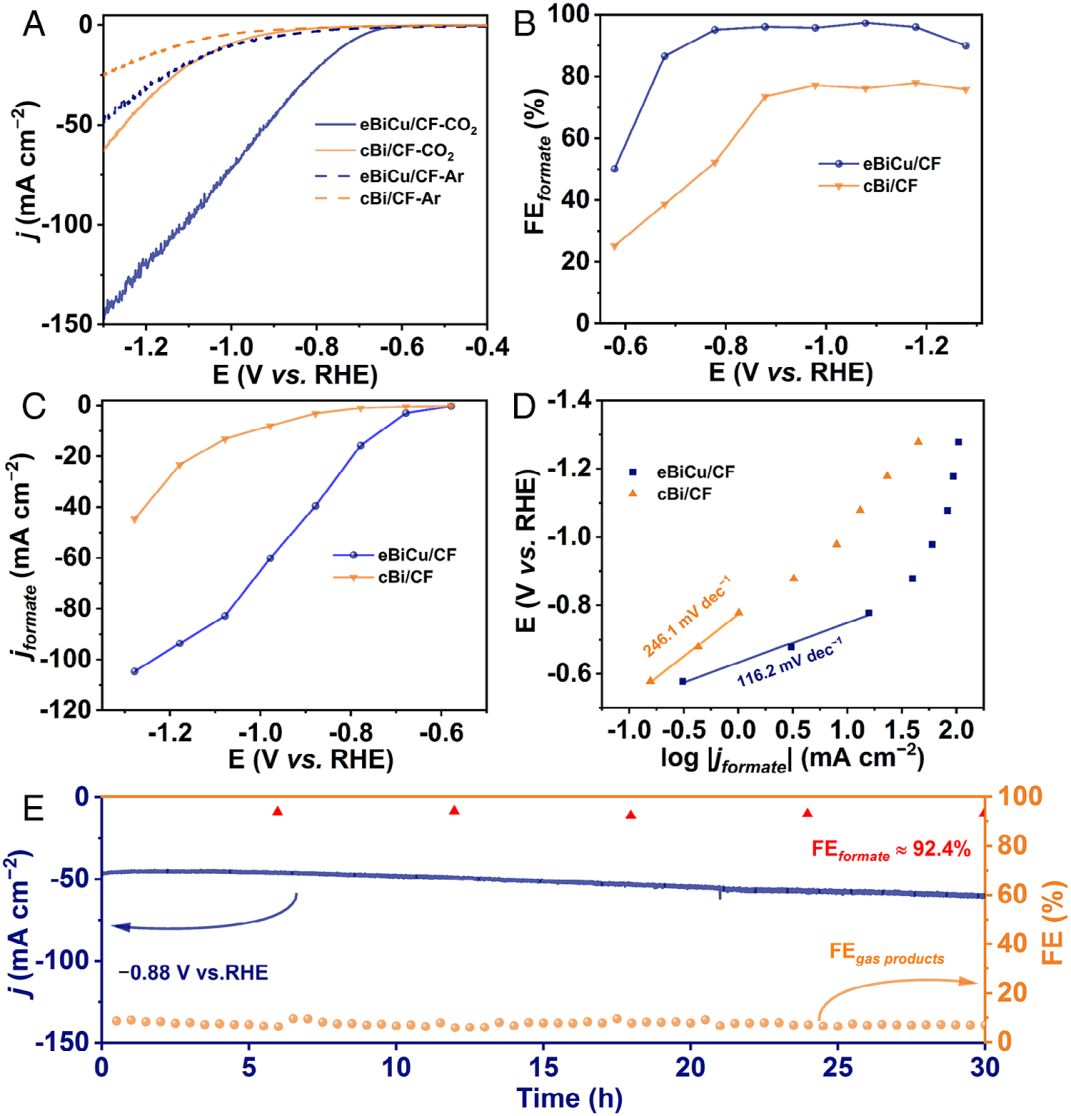
Electrochemical CO_2_RR performance of eBiCu/CF: A, Linear sweep voltammogram (LSV) curves, B, faradaic efficiency for formate (FE_formate_), C, *j*_formate_, D, Tafel plots, and E, long‐term stability.

Furthermore, to decode the advantages of bimetallic synergy, we compared the electrochemical CO_2_RR performance of eBiCu/CF with that of the commercial Bi powder‐coated CF electrode, namely, cBi/CF. It is evident from Figure 2A that the eBiCu/CF electrode exhibits a higher current density and a smaller initial potential, indicating superior electrocatalytic performance and highlighting the structural benefits of the self‐supported array catalyst. We also conducted potentiostatic electrolysis to analyze the product distribution, and the *i–t* curves are shown in Figure S9. Concretely, the current densities of the cBi/CF electrode exhibit significant fluctuation and are much lower than those of eBiCu/CF. As shown in Figure 2B, the FE_formate_ of the eBiCu/CF electrode exceeds 90% in the potential range of −0.78 to −1.18 V and reaches a maximum of 97.2% at −1.08 V, which is much higher than that of the cBi/CF electrode. In addition, the *j*_formate_ of the electrocatalysts at different potentials was calculated (Figure 2C), and the results show that the *j*_formate_ values of eBiCu/CF are much higher than those of cBi/CF, reaching 104.6 mA cm^−2^ at −1.28 V, further demonstrating the excellent activity of eBiCu/CF for electrocatalytic CO_2_RR to produce formate. The reaction kinetics of electrochemical CO_2_RR were analyzed by the Tafel slope. Figure 2D shows that the Tafel slope of eBiCu/CF (116.2 mV dec^−1^) is much smaller than that of the cBi/CF electrode (246.1 mV dec^−1^), indicating the excellent reaction kinetics of eBiCu/CF and facilitating the catalytic reaction. In addition, the long‐term stability test was carried out at −0.88 V (Figure 2E) and showed that the eBiCu/CF catalyst maintained stable current density and FE*_formate_* during 30 h of electrolysis, demonstrating its excellent electrochemical CO_2_RR stability compared with the state‐of‐the‐art counterparts (Table S1).

The double‐layer capacitance (C_dl_) can be used to estimate the electrochemical active surface area (ECSA) of the catalysts to further evaluate the inherent activity. Figure S10A,B shows the CV curves of the catalysts at different scan rates within the non‐faradaic potential range, and a C_dl_ of 24.6 mF cm^−2^ was obtained for the eBiCu/CF catalyst through further calculations, which is 35 times greater than that of the cBi/CF catalyst (0.7 mF cm^−2^; Figure S10C). This indicates that the self‐supported nanoarray superstructure of the eBiCu/CF catalyst endows it with abundant catalytic active sites. As shown in Figure S10D, the half‐circle diameter of eBiCu/CF is much smaller than that of cBi/CF, indicating a faster rate of charge transfer. Based on the electrochemical tests conducted, it can be concluded that the self‐supported eBiCu/CF nanoarray electrode exhibits significantly higher catalytic activity and selectivity when compared to the other contrast electrodes. This can be attributed to various factors including, (a) the 3D porous and open network of interconnected array superstructure, which promotes efficient charge and mass transfer; (b) the formation of electron‐rich Bi resulting from electronic transfer from Cu to Bi, which exhibits excellent intrinsic catalytic activity for electrochemical CO_2_RR to produce formate; (c) the self‐supported catalyst without the use of binders, featuring high conductivity and preventing adsorption sites and catalytic active sites from being covered; (d) the porous structure of the nanosheets, which can provide rich structural defects and edge sites that possess the higher intrinsic activity and contribute to improved electrocatalytic performance.

### 2.3 Characterizations and discussion of Cu_x_Ni_1−x_(OH)_2_/CF

**FIGURE 3:**
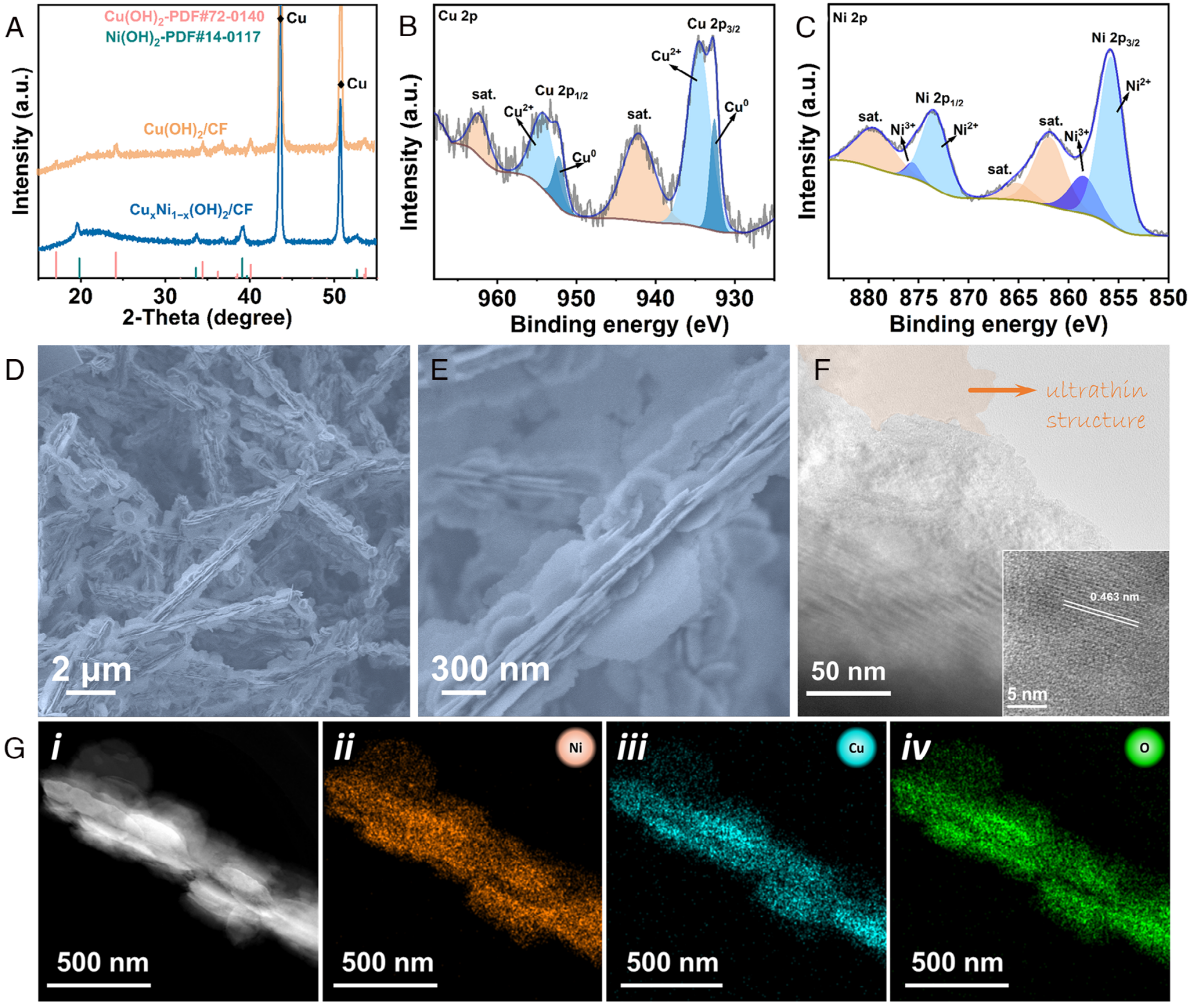
Characterizations of Cu_x_Ni_1−x_(OH)_2_/CF: A, X‐ray diffraction (XRD) pattern, x‐ray photoelectron spectroscopy (XPS) spectra for B, Cu 2p and C, Ni 2p, D,E, SEM, F, transmission electron microscopy (TEM), and G, energy‐dispersive x‐ray (EDX) elemental mapping images (note: for (F), pseudo‐color is used to highlight the ultrathin structure of the nanosheet).

According to the characteristic of anodic CHOR, we further developed Cu_x_Ni_1−x_(OH)_2_/CF electrocatalyst through hydrothermal treatment of Cu(OH)_2_/CF nanoarray. The XRD patterns in Figure 3A show that the diffraction peaks of Cu(OH)_2_ disappear while the diffraction peaks of Ni(OH)_2_ appear, suggesting the possible formation of Cu‐doped Ni(OH)_2_ phase. The electronic structure of Cu_x_Ni_1−x_(OH)_2_/CF was investigated by XPS in detail. As shown in Figure 3B, the peaks of Cu 2p_1/2_ at 954.4 and 952.3 eV correspond to Cu^2+^ and Cu^0^, respectively. The Cu 2p_3/2_ signal can also be fitted into two peaks at 934.5 and 932.6 eV, which are attributed to Cu^2+^ and Cu^0^, respectively.^[^ ^16^ ^]^ The Ni 2p spectrum shows two main peaks of Ni 2p_1/2_ (856.0 eV) and Ni 2p_3/2_ (873.6 eV) for Ni^2+^ (Figure 3C). Meanwhile, the two peaks at 875.7 and 858.6 eV belonging to Ni^3+^ might be caused by partial surface oxidation, which has been widely observed in other transition metal‐based compounds.^[^ ^17^ ^]^ In addition, the O 1s spectrum also shows the existence of three kinds of oxygen in the sample (Figure S11), indicating the integration of bimetallic hydroxides. The SEM images reveal that the morphology of the nanowire array is maintained after hydrothermal casting, while tightly stacked and connected ultrathin nanosheets are in situ formed on the surface, making the surface of the nanowires rougher and further increasing the surface area of Cu_x_Ni_1−x_(OH)_2_/CF (Figure 3D,E). The TEM images also confirm the nanosheet‐on‐nanowire morphology of Cu_x_Ni_1−x_(OH)_2_/CF, and the ultrathin nanostructure of the nanosheets is identified (Figure 3F). Furthermore, the HRTEM image displays a crystal lattice pattern of 0.463 nm, corresponding to the (001) crystal plane of Ni(OH)_2_ (0.461 nm), and the slight deviation may be due to the lattice distortion caused by Cu doping in the Ni(OH)_2_ lattice (Figure 3F, inset). The EDX mapping images show a uniform distribution of Ni, Cu, and O throughout the entire nanowire, further indicating the uniform growth of the nanosheet‐wrapped nanowire (Figure 3G). In particular, Figure S12 shows that the elemental content ratio of (Ni+Cu) to O is close to 1:2, which is consistent with the XRD result, proving the successful construction of the designed electrocatalyst.

### 2.4 Electrochemical performance of Cu_x_Ni_1−x_(OH)_2_/CF for CHOR

**FIGURE 4:**
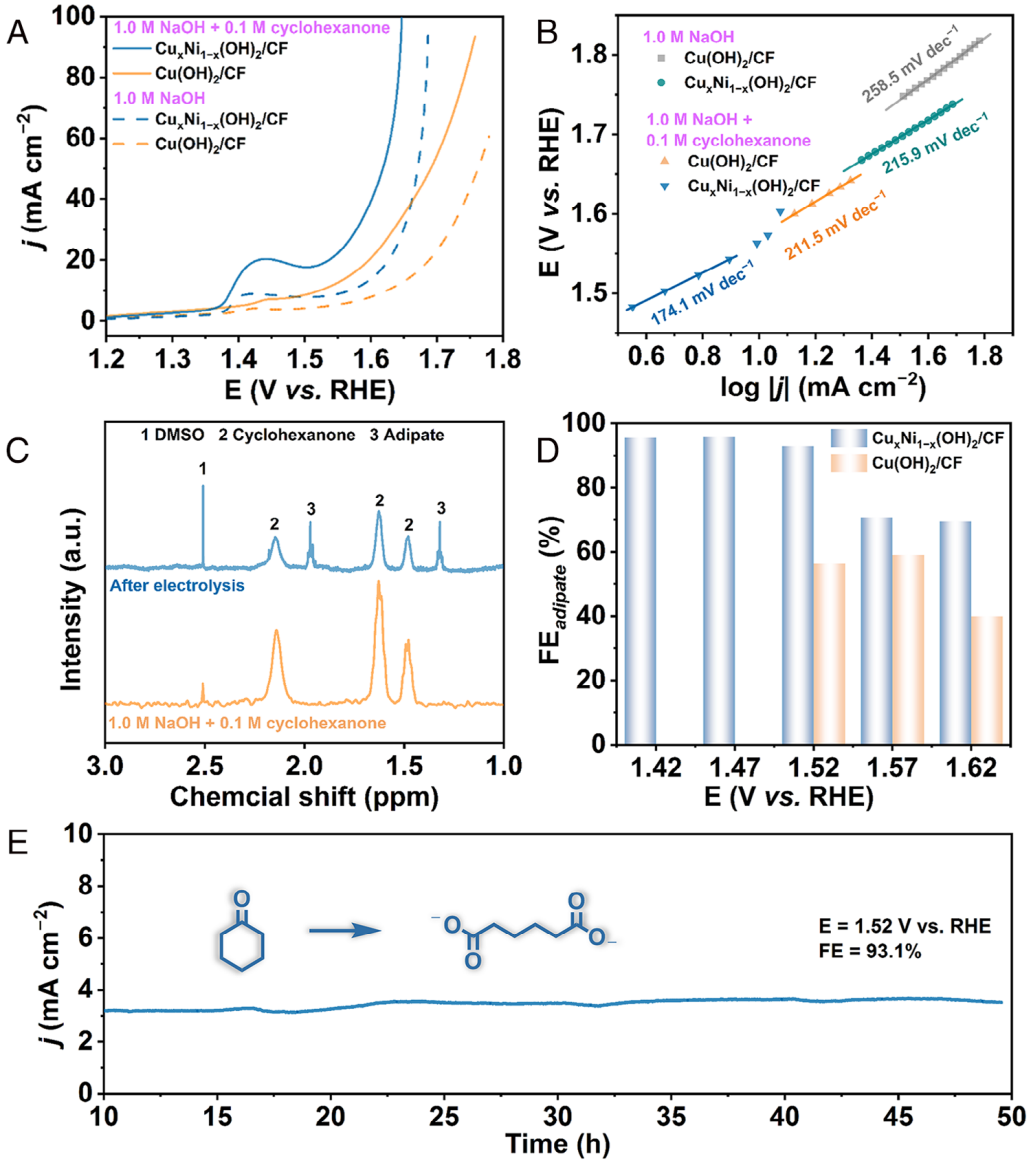
Electrochemical cyclohexanone oxidation reaction (CHOR) performance of Cu_x_Ni_1−x_(OH)_2_/CF: A, Linear sweep voltammogram (LSV) curves, B, Tafel plots, C, ^1^H NMR spectra, D, faradaic efficiency of adipate (FE_adipate_), and E, long‐term stability.

The electrocatalytic performance of Cu_x_Ni_1−x_(OH)_2_/CF for CHOR was evaluated in a standard three‐electrode system. Figure 4A shows the LSV curves of the prepared electrode in 1.0 M NaOH solution with or without 0.1 M cyclohexanone added (90% iR compensation correction). Notably, the electrochemical CHOR performance of Cu_x_Ni_1−x_(OH)_2_/CF is superior to that of Cu(OH)_2_/CF. The initial potential of Cu_x_Ni_1−x_(OH)_2_/CF is significantly lowered after the addition of 0.1 M cyclohexanone. At the same time, the Tafel slope is significantly reduced upon the addition of cyclohexanone, indicating that CHOR has more favorable thermodynamics and faster catalytic reaction kinetics compared to OER (Figure 4B). Subsequently, the products during CHOR were quantitatively analyzed using potentiostatic electrolysis, and it can be seen from the *i–t* curves that the current densities of Cu_x_Ni_1−x_(OH)_2_/CF are significantly higher than those of Cu(OH)_2_/CF at the same potentials (Figure S13). The ^1^H NMR spectrum confirms the formation of adipate (Figure 4C). As shown in Figure 4D, FE_adipate_ of Cu_x_Ni_1−x_(OH)_2_/CF is much higher than those of Cu(OH)_2_/CF, suggesting the significant advantage of a bimetallic array in electrochemical CHOR. Notably, the FE_adipate_ of Cu_x_Ni_1−x_(OH)_2_/CF is close to 100% at the potentials below 1.52 V. However, at the higher potentials, the FE_adipate_ gradually decreases, which is attributed to the competitive OER reaction. At the same time, the ECSAs of Cu_x_Ni_1−x_(OH)_2_/CF and Cu(OH)_2_/CF were measured, and the results show that the ECSA of Cu_x_Ni_1−x_(OH)_2_/CF is much larger than that of Cu(OH)_2_/CF, indicating that the hierarchical surface of Cu_x_Ni_1−x_(OH)_2_/CF can provide more accessible active sites (Figure S14A–C). Further electrochemical impedance spectroscopy (EIS) test shows that Cu_x_Ni_1−x_(OH)_2_/CF has superior charge transfer kinetics (Figure S14D). In addition, the long‐term stability was executed, and the result is shown in Figure 4E. During nearly 50 h of electrolysis, Cu_x_Ni_1−x_(OH)_2_/CF was able to maintain a stable current density while achieving a high FE_adipate_ of 93.1%, superior to the reported performances (Table S2). In conclusion, through bimetallic construction, the designed Cu_x_Ni_1−x_(OH)_2_/CF has excellent performance for the oxidation of cyclohexanone to adipate.

### 2.5 Integration of CO_2_RR and CHOR for coupling electrosynthesis

**FIGURE 5:**
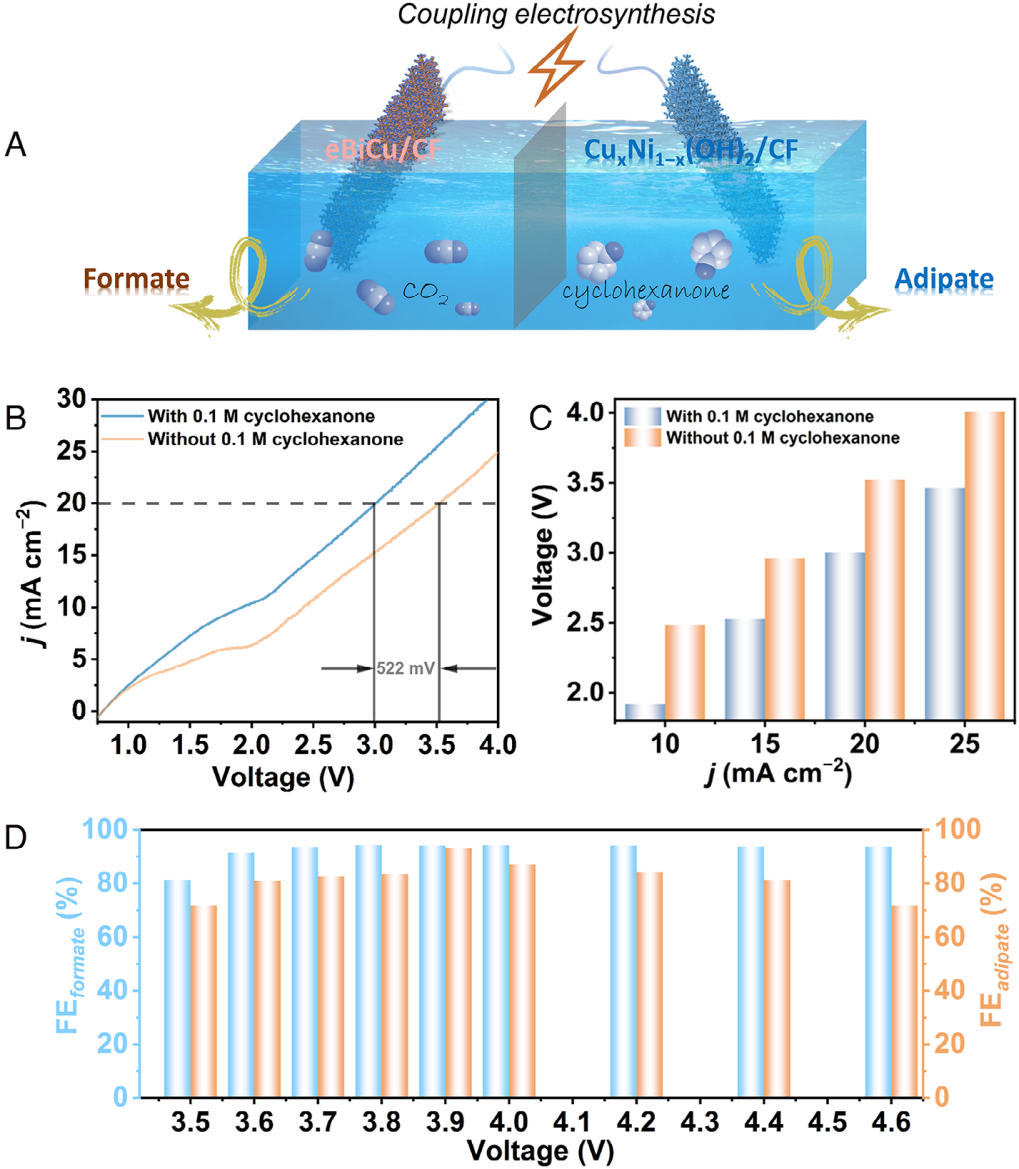
Two‐electrode electrolyzer for coupling electrosynthesis of formate and adipate using eBiCu/CF as the cathodic catalyst and Cu_x_Ni_1−x_(OH)_2_/CF as the anodic catalyst: A, schematic illustration, B, linear sweep voltammogram (LSV) curves, C, potentials at different current densities, and D, faradaic efficiencies (FEs).

Given the excellent electrochemical performance of eBiCu/CF for CO_2_RR and Cu_x_Ni_1−x_(OH)_2_/CF for CHOR, a bipolar membrane (BPM) separated CO_2_RR//CHOR full cell was constructed (Figure 5A). The catholyte was CO_2_‐saturated 0.5 M KHCO_3_ aqueous solution, while the anolyte was 1.0 M NaOH solution with or without the addition of 0.1 M cyclohexanone. When the anode contained 0.1 M cyclohexanone, the electrolysis cell only required 3.0 V to achieve a current density of 20 mA cm^−2^, which is 522 mV lower than the potential required without cyclohexanone (Figure 5B). At the same current density, the electrolysis voltage of the CO_2_RR//CHOR electrolysis cell was much lower than that of the CO_2_RR//OER cell, highlighting the significant thermodynamic advantage of CHOR over OER (Figure 5C). Further investigation of the products at the anode and cathode was conducted using potentiostatic electrolysis, and the *i–t* curves are shown in Figure S15, demonstrating good stability during the electrolysis process. More importantly, Figure 5D shows that both the anode and cathode maintained high FEs in a wide potential range, highlighting the vista of coupling electrosynthesis. Moreover, the XRD and SEM results of the electrolyzed samples also confirm their exceptional structure stability (Figures S16 and S17). The above results multidimensionally indicate that the CO_2_RR//CHOR electrolysis system is feasible for the coupling electrosynthesis of formate and adipate.

## 3 CONCLUSIONS

This work systematically demonstrated the use of Cu(OH)_2_ nanowire arrays as a processable material platform to construct self‐supported bimetallic array superstructures with multiple structural advantages for highly efficient coupling electrosynthesis. By virtue of the self‐supported nanosheet array superstructure with abundant accessible active sites, high conductivity, and fast mass transport, the eBiCu/CF cathodic catalyst for electrochemical CO_2_‐to‐formate conversion can achieve over 90% FE_formate_ in the wide potential range of 400 mV, and *j*_formate_ can reach up to 104.6 mA cm^−2^ at −1.28 V. Meanwhile, using the nanosheet‐on‐nanowire array superstructured Cu_x_Ni_1−x_(OH)_2_/CF as the anode for adipate electrosynthesis, nearly 100% FE_adipate_ was obtained, and the current densities are much higher than those of OER. More reliably, the CO_2_RR//CHOR electrolyzer requires a voltage of only 3.0 V to achieve the current density of 20 mA cm^−2^, which is 522 mV lower than that required by CO_2_RR//OER, and the maximal FE_formate_ and FE_adipate_ can reach up to 94.2% and 93.1%, respectively. The coupling electrolysis system can significantly improve the economic benefit of CO_2_ electrolysis and simultaneously achieve the high‐efficiency electrosynthesis of adipate, providing valuable references for the green synthesis of other high‐value‐added chemical products.

## 4 EXPERIMENTAL SECTION

### 4.1 Preparation of catalysts

#### 4.1.1 Preparation of Cu(OH)_2_/CF

Cu(OH)_2_/CF was synthesized according to our previous work.^[^ ^18^ ^]^ The obtained mazarine Cu(OH)_2_/CF was stored in a vacuum desiccator at room temperature.

#### 4.1.2 Preparation of Bi‐O‐Cu/CF

1.0 mmol of Bi(NO_3_)_3_·5H_2_O was dissolved in a solution consisting of 12 mL of H_2_O, 3 mL of glycerol, and 5 mL of N,N‐dimethyl formamide (DMF) through vigorous stirring for 30 min. The mixed solution was then transferred to a 50 mL Teflon‐lined autoclave, where a piece of Cu(OH)_2_/CF with a size of 2 × 2.5 cm^2^ was added, sealed, and heated at 120°C for 12 h. After the reaction, the obtained Bi‐O‐Cu/CF was washed with deionized water and ethanol several times and dried in a vacuum at 60°C.

#### 4.1.3 Preparation of eBiCu/CF

eBiCu/CF was prepared by in situ electrochemical conversion of Bi‐O‐Cu/CF in a three‐electrode system consisting of a CO_2_‐saturated 0.5 M KHCO_3_ solution. Concretely, 100 cycles of cyclic voltammetry (CV) experiments were carried out at a scanning rate of 100 mV s^−1^ within the potential range of −0.8 to −1.8 V (vs. Ag/AgCl). The as‐obtained electrode was then taken out from the electrolyte, rinsed with deionized water, and directly used as a working electrode for subsequent experiments.

#### 4.1.4 Preparation of cBi/CF

1.0 mg of commercial Bi powder was dispersed in a 100 μL mixed solution consisting of H_2_O (70 μL), isopropanol (20 μL), and Nafion (10 μL) with ultrasonic treatment for 2 h. Then, the above ink was dropped onto the CF with the size of 1.0 × 1.0 cm^2^ and dried naturally at room temperature.

#### 4.1.5 Preparation of Cu_x_Ni_1−x_(OH)_2_/CF

0.436 g of Ni(NO_3_)_2_·6H_2_O, 0.154 g of NH_4_F, and 0.997 g of urea were dissolved in 40 mL of H_2_O and stirred for 30 min to get a homogeneous solution. The mixed solution was then transferred to a 100‐mL Teflon‐lined autoclave, where a piece of Cu(OH)_2_/CF with a size of 2 × 2.5 cm^2^ was added, sealed, and heated at 100°C for 10 h. After the reaction, the obtained Cu_x_Ni_1−x_(OH)_2_/CF was taken out from the solution, washed with deionized water and ethanol several times, and dried in a vacuum at 60°C.

### 4.2 Electrochemical measurements

Other experimental details are provided in the Supporting Information.

## AUTHOR CONTRIBUTIONS

Dong‐Dong Ma and Qi‐Long Zhu conceived the research and designed the experiments. Li Liu and Yingchun He carried out the synthesis, material characterizations, and electrochemical measurements. Li Liu, Yingchun He, Qing Li, Changsheng Cao, Dong‐Dong Ma, Xin‐Tao Wu, and Qi‐Long Zhu analyzed and discussed the data. Li Liu, Yingchun He, Dong‐Dong Ma, and Qi‐Long Zhu drafted the manuscript. All authors discussed and revised the manuscript.

## CONFLICT OF INTEREST STATEMENT

The authors declare no conflicts of interest.

## Supporting information

Supporting Information

## Data Availability

All data of this work are present in the article and Supporting Information. The other data that support the findings of this work are available from the corresponding author upon reasonable request.
