# Supplementary material for: Self‐supported bimetallic array superstructures for high‐performance coupling electrosynthesis of formate and adipate
Source: Exploration (Beijing). 2023 Dec 21;4(3):20230043. doi: 10.1002/EXP.20230043 (PMC11189569; doi:10.1002/EXP.20230043)
Supplement: Supplementary file 1 — Supporting Information [file EXP2-4-20230043-s001.docx]

Supporting Information for

**Self-supported bimetallic array superstructures for high-performance coupling electrosynthesis of formate and adipate**

Li Liu^#,1,3^, Yingchun He^#,1,3^, Qing Li^1,3^, Changsheng Cao^1^, Minghong Huang^4^, Dong-Dong Ma*^,1,3^, Xin-Tao Wu^1,2,3^, Qi-Long Zhu*^,1,2,3^

^1^ State Key Laboratory of Structural Chemistry, Fujian Institute of Research on the Structure of Matter, Chinese Academy of Sciences, Fuzhou 350002, China

^2^ Fujian Science & Technology Innovation Laboratory for Optoelectronic Information of China, Fuzhou 350108, China

^3^ University of Chinese Academy of Sciences, Beijing 100049, China

^4^ School of Civil and Environmental Engineering, University of Technology Sydney, Ultimo, New South Wales 2007, Australia

E-mail: [madong@fjirsm.ac.cn](mailto:madong@fjirsm.ac.cn) (Dong-Dong Ma), [qlzhu@fjirsm.ac.cn](mailto:qlzhu@fjirsm.ac.cn) (Qi-Long Zhu).

^#^ These authors contributed equally to this work.

**Experimental Section**

**Chemicals and materials**

Bismuth nitrate pentahydrate (Bi(NO_3_)·5H_2_O, ≥99.0%), urea (CH_4_N_2_O, ≥99.0%), nickel nitrate hexahydrate (Ni(NO_3_)_2_·6H_2_O, ≥98.0%), glycerol (Gly, ≥99.0%), sodium hydroxide (NaOH, ≥96.0%), ammonium persulfate ((NH_4_)_2_S_2_O_8_, ≥98.0%), N,Nʹ-dimethylformamide (DMF, ≥99.5%), hydrochloric acid (HCl, 36.0~38.0%), acetone (≥99.5%), ammonium fluoride (NH_4_F, ≥96.0%), potassium bicarbonate (KHCO_3_, ≥99.5%), dimethylsulfoxide (DMSO, 99.8%) were purchased from Sinopharm Chemical Reagent Co., Ltd. (China). Bismuth (Bi, ≥99.99%) and cyclohexanone (C_6_H_10_O, ≥99.5%) was purchased from Shanghai Macklin Biochemical Co., Ltd. (China). Deuterium Oxide (D_2_O, (D, 99.9%)) was purchased from Shanghai Titan Technology Co., Ltd. (China). Cu foam (CF) was purchased from Tianjin Annuohe New Energy Technology Co., Ltd. (China). Bipolar membrane (BMP) was purchased from Beijing Tingrun Membrane Technology Development Co., Ltd. (China). The deionized water (18.2 MΩ cm) used in all tests was generated by passing through an ultra-pure purified water system.

**Materials characterization**

The morphologies of the samples were investigated with the scanning electron microscope (SEM, JSM6700-F) and transmission electron microscope (TEM, FEI titan themis 200). X-ray diffraction (XRD) patterns of the samples were obtained by Rigaku MiniFlex 600 at 40 kV voltage and 15 mA current using Cu Kα-radiation. The electronic states of elements were examined using X-ray photoelectron spectroscopy (XPS, Thermo Fisher ESCALAB 250Xi), using the C 1s peak (binding energy = 284.8 eV) as the internal reference. ^1^H NMR spectra were performed on the ECZ400S spectrometer (400 MHz). The gas products were continuously conveyed into the gas-sampling loop (250 μL) of a gas chromatograph (GC, Agilent 7820A) for analyzing the gas products using thermal conductivity detector (TCD) and flame ionization detector (FID). The GC calibration curves for H_2_ and CO were fitted by five times independent single point sampling.

**Electrochemical measurements**

All the electrochemical measurements were carried out on the CHI760E electrochemical workstation (Shanghai Chen-Hua Instrument Corporation, China) with a typical three-electrode cell at room temperature. Moreover, saturated Ag/AgCl and Pt mesh were used as the reference electrode and counter electrode, respectively. Electrochemical CO_2_RR was performed in an H-type electrochemical cell separated by a proton exchange membrane (Nafion117) using CO_2_-saturated 0.5 M KHCO_3_ solution (pH = 7.2) as the electrolyte. The flow of CO_2_ (99.999%) or Ar (≥99.999%) was 20 mL min^−1^ (using Mass flow controller D07-7B) during the electrolysis. The electrosynthesis of adipic acid was tested using a single cell without a membrane, and the electrolyte was 1.0 M NaOH solution with 0.1 M cyclohexanone (pH = 14). The magnet speed is 1000 rpm to increase the dispersion of cyclohexanone in the electrolyte. All samples for test were cut into 1.0 × 1.0 cm^2^ and directly used as working electrodes. All electrochemical measurements of potentials were calibrated using the following equation: E_RHE_ = E_Ag/AgCl_ + 0.197 + 0.059 × pH (V). Linear sweep voltammetry (LSV) curves were obtained at a scanning rate of 5 mV s^−1^ in different electrolytes. Faradaic efficiencies (FEs) for formate were calculated by the equation: FE = nNF/Q, where n is the mole of product, N is 2, F is the Faradaic constant (96,485 C mol^−1^), Q is the total charge passed through the working electrode. The mole numbers of the adipate (The product exists in the form of adipate rather than adipic acid since the product is not acidified) were calculated by the following equation: n_adipate_/n_DMSO_=A_adipate_N_DMSO_/A_DMSO_N_adipate_, where A_adipate_ is the integral area of the adipate peak in the NMR spectrum, A_DMSO_ is the integral area of the DMSO peak, N_adipate_ is the H number of the adipate peak, N_DMSO_ is the H number of the DMSO peak, and n_DMSO_ is the mole number of the DMSO internal standard. Tafel plots were extrapolated from the corresponding linear region of overpotential versus the logarithm of current density. Electrochemical impedance spectra (EIS) were obtained at a frequency ranging from 0.01 to 10^5^ Hz with an AC amplitude of 5.0 mV. Electrochemically active surface areas (ECSAs) were evaluated by conducting CV tests at different scan rates in the non-faradaic region. CO_2_RR//CHOR full cell was tested used a bipolar membrane to separate the anode and cathode. eBiCu/CF and Cu_x_Ni_1−x_(OH)_2_/CF were used as the cathode and anode, respectively. CO_2_-saturated 0.5 M KHCO_3_ was used as the catholyte, 1.0 M NaOH + 0.1 M cyclohexanone was used as the anolyte.


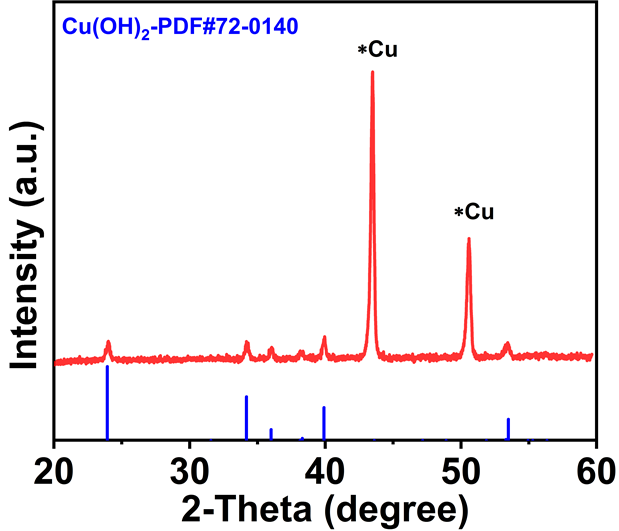


**FIGURE S1.** XRD pattern of Cu(OH)_2_/CF.


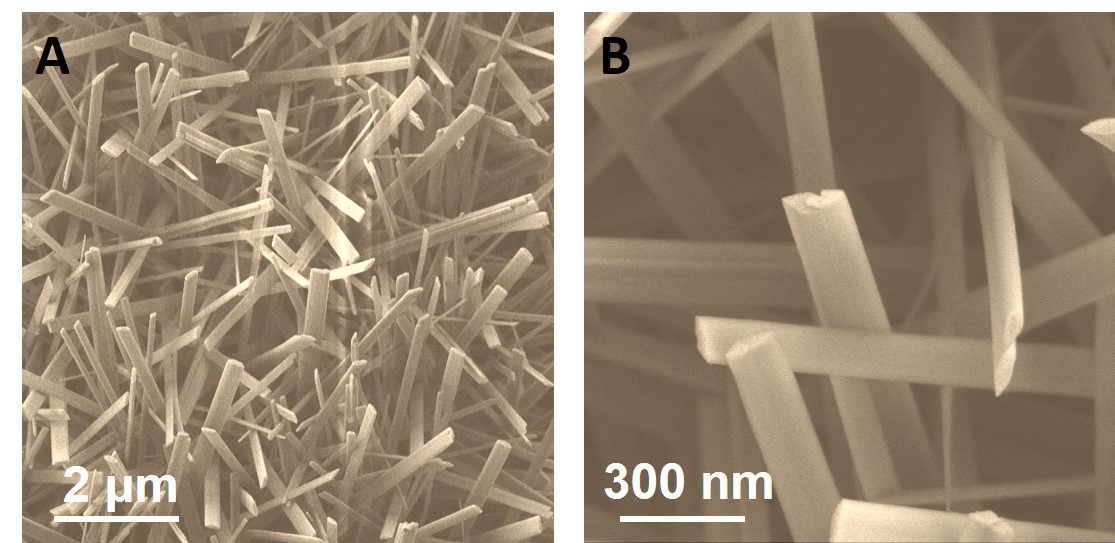


**FIGURE S2.** SEM images of Cu(OH)_2_/CF.


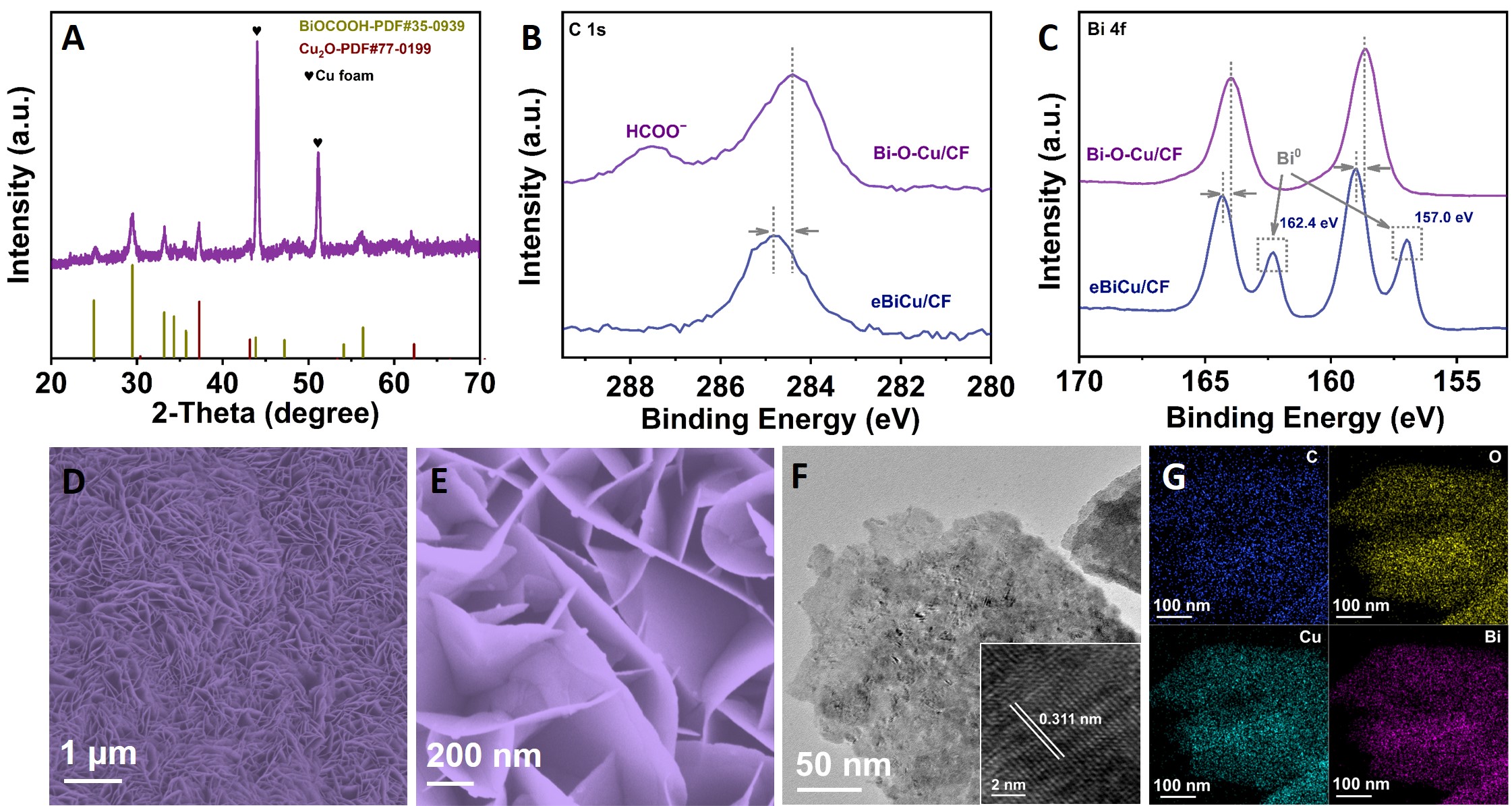


**FIGURE S3.** Characterizations of Bi-O-Cu/CF: (A) XRD pattern, XPS spectra for (B) C 1s and (C) Bi 4f, (D, E) SEM images, (F) TEM and (G) EDX elemental mapping images.

The SEM images reveal that Bi-O-Cu/CF is composed of an array structure of ultrathin nanosheets. The TEM image also confirms the morphology of ultrathin Bi-O-Cu/CF nanosheets. The lattice fringe of 0.311 nm in the HRTEM image corresponds to the (102) crystal face of BiOCOOH, which further confirms the XRD results. The EDX image shows the uniform distribution of C, O, Cu, and Bi throughout the nanosheet, indicating the uniform growth of Bi-O-Cu/CF.


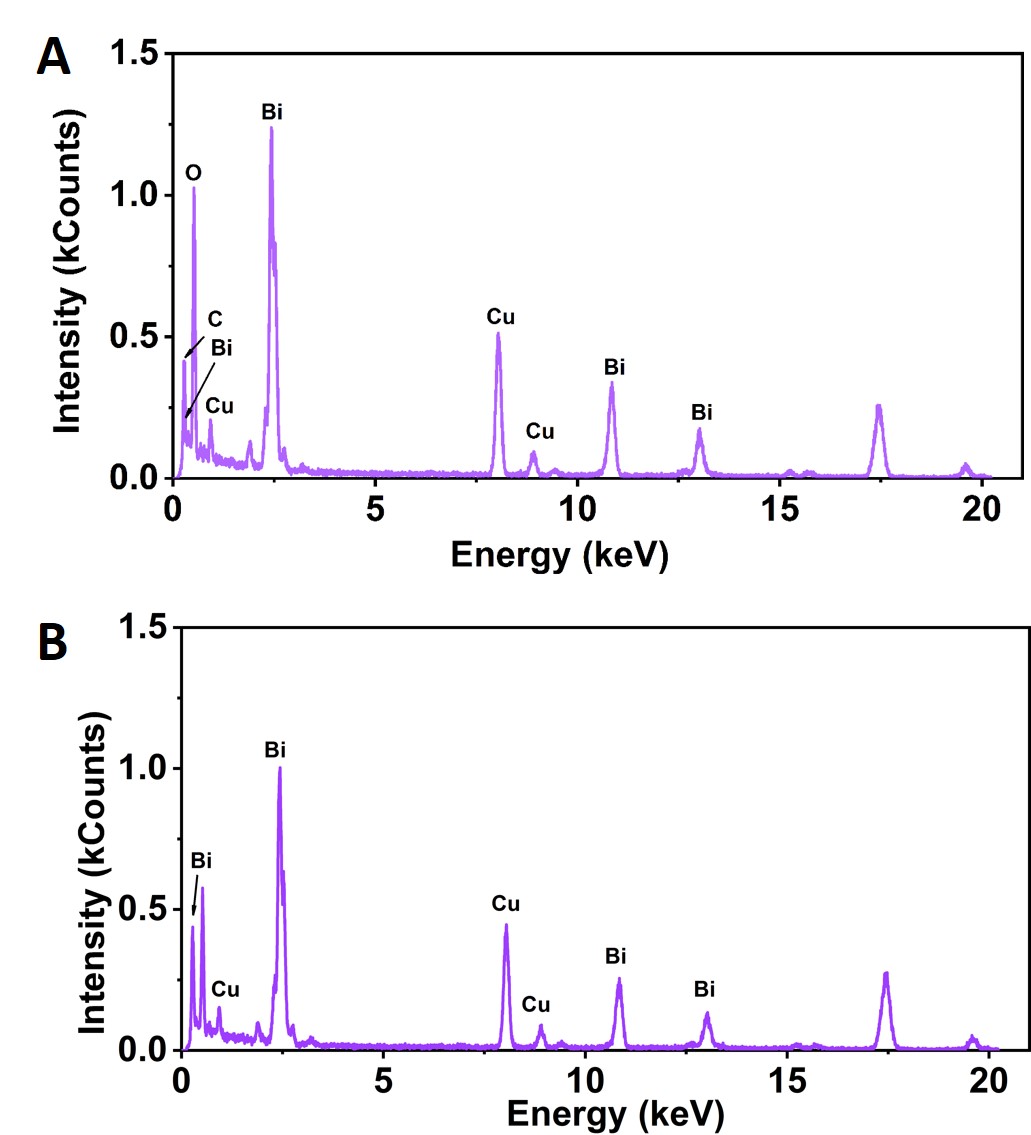


**FIGURE S4.** The elemental EDX results of (a) Bi-O-Cu/CF and (b) eBiCu/CF.


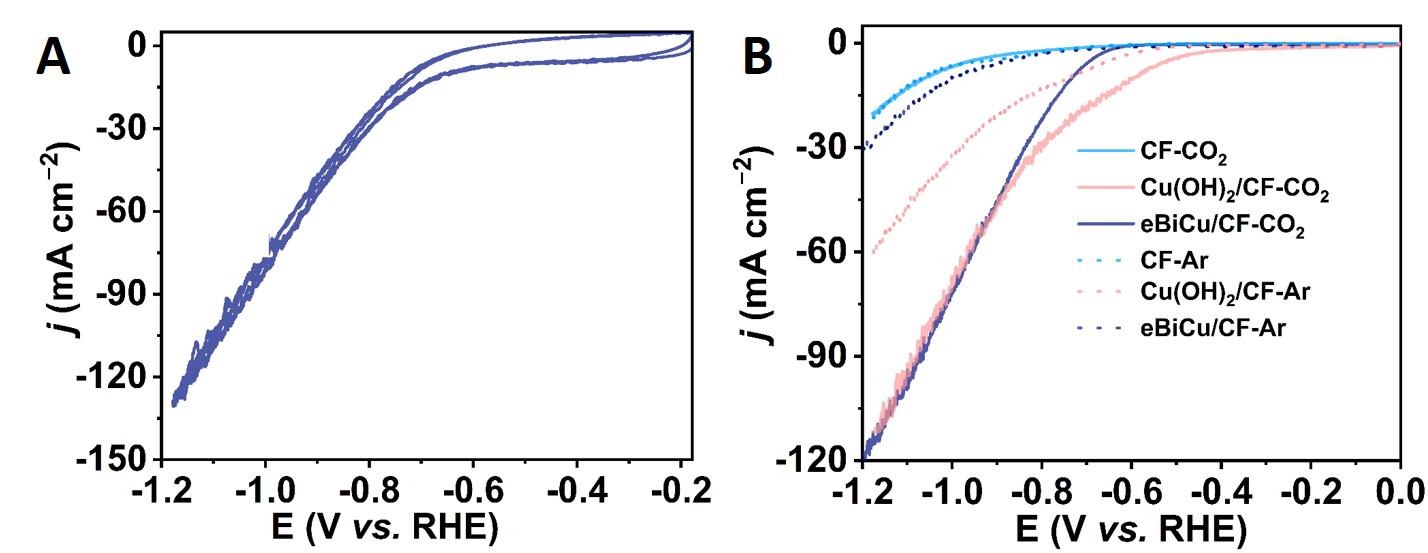


**FIGURE S5.** (A) The 3-cycle CV curves of Bi-O-Cu/CF after 100 cycles and (B) LSV curves of CF, Cu(OH)_2_/CF and eBiCu/CF.

Fig. S5A shows the 3 cycles of CV curves of Bi-O-Cu/CF after 100 cycles, which overlap completely, indicating that Bi-O-Cu/CF has been fully reduced and converted to eBiCu/CF. The obtained eBiCu/CF was directly used as the working electrode for subsequent CO_2_RR test.


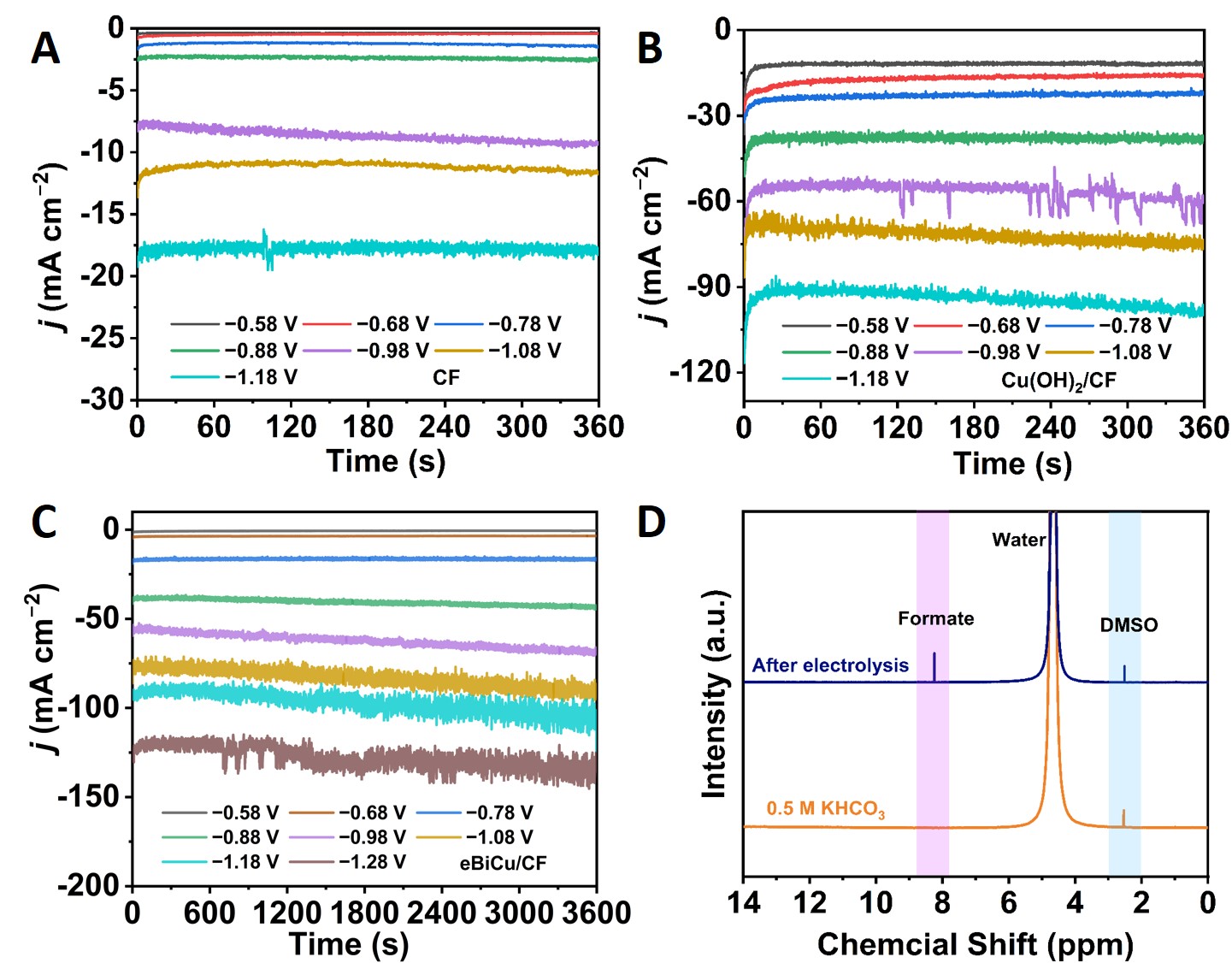


**FIGURE S****6.** The *i-t* curves for (A) CF, (B) Cu(OH)_2_/CF, and (C) eBiCu/CF; (D) ^1^H NMR spectra for eBiCu/CF after electrolysis.

**FIGURE S7.** Comparison of FEs at different potentials for CF, Cu(OH)_2_/CF and eBiCu/CF.


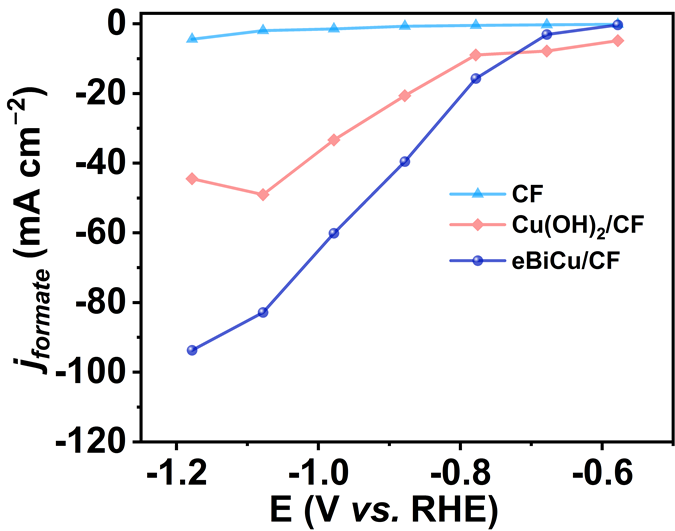


**FIGURE S8.** Partial current density of formate for CF, Cu(OH)_2_/CF and eBiCu/CF.


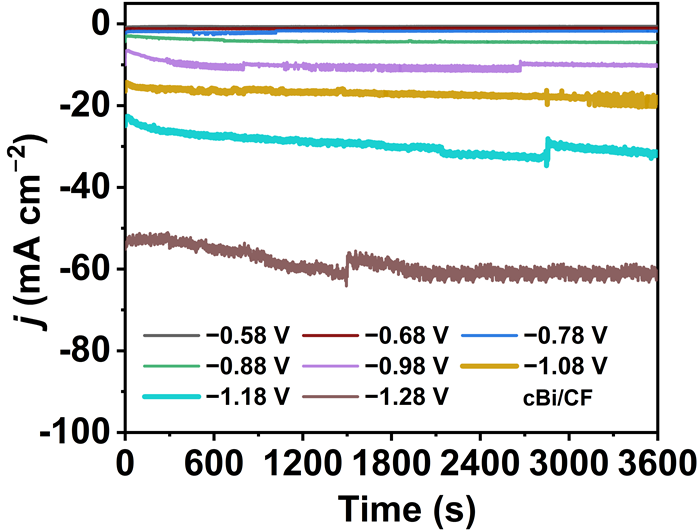


**FIGURE S9.** *I-t* curves for cBi/CF.


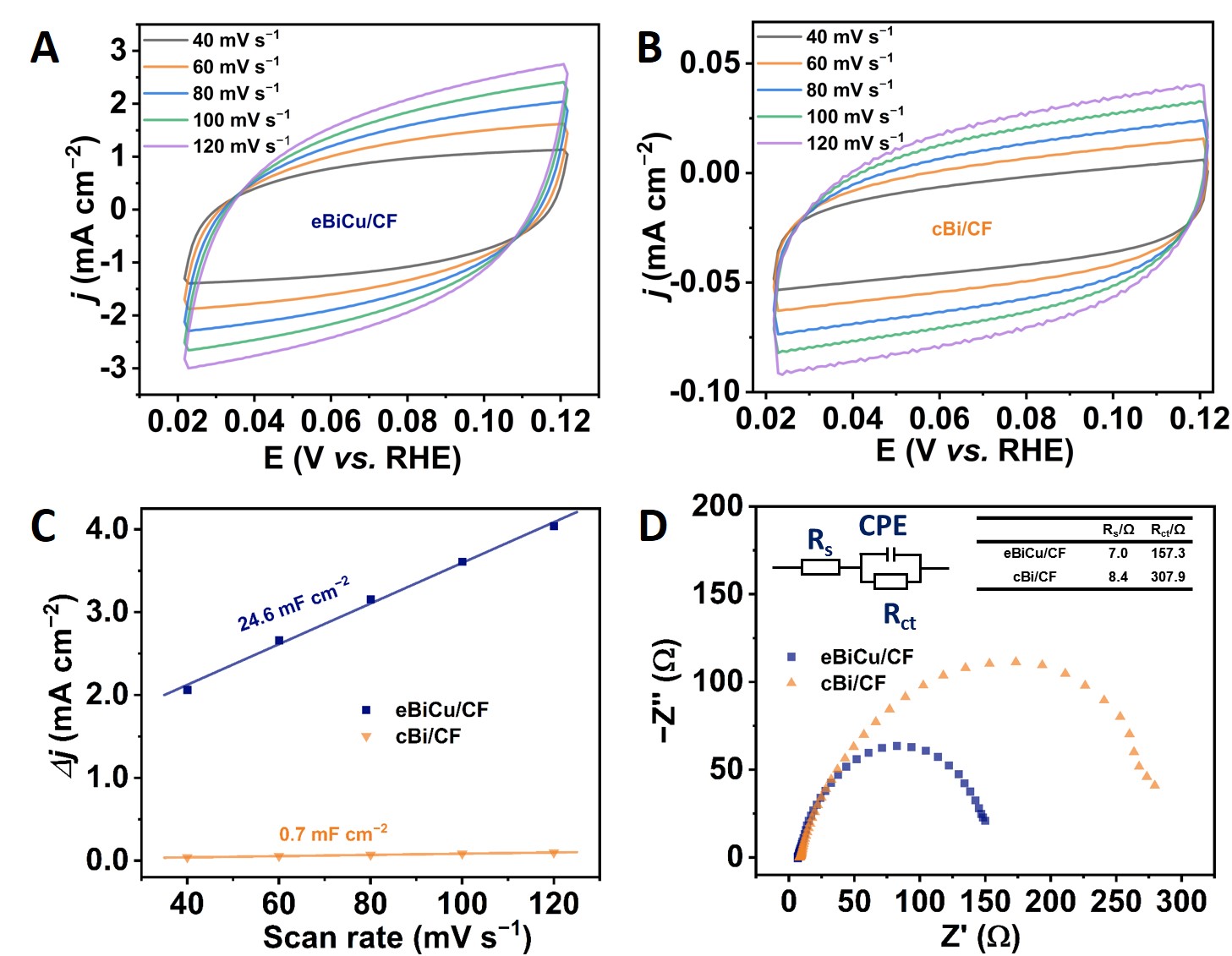


**FIGURE S10.** CV curves of (A) eBiCu/CF and (B) cBi/CF at different scan rates; (C) capacitances inferred from charge current density diﬀerences against scan rates; (D) EIS plots.


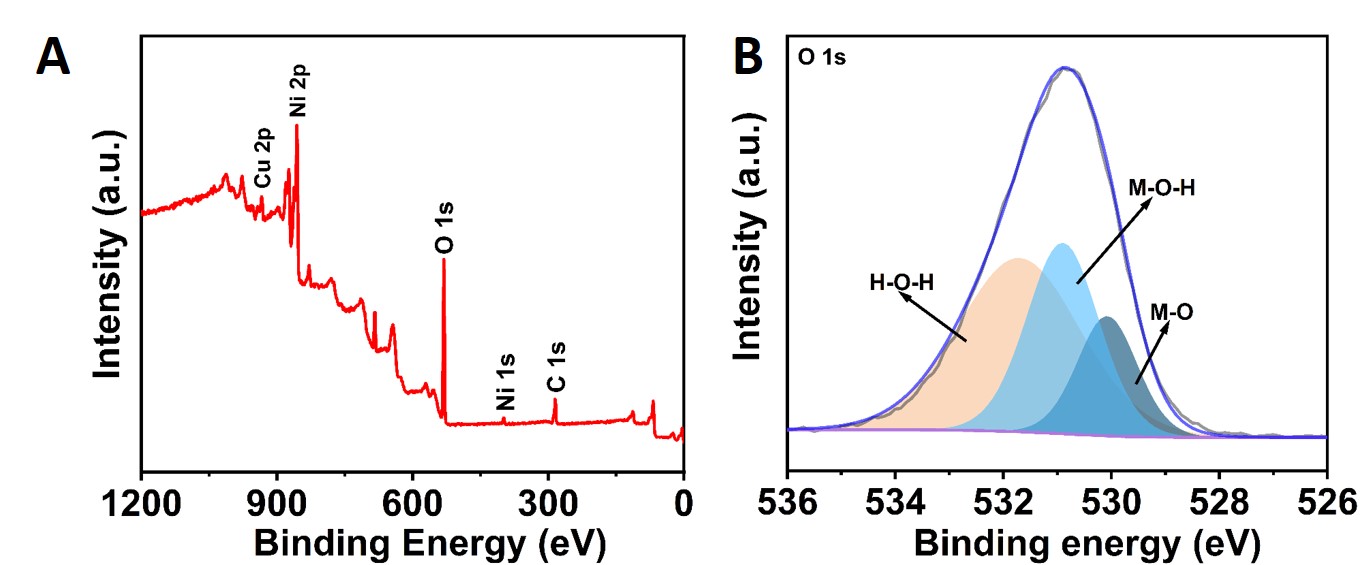


**FIGURE S11.** XPS spectra of Cu_x_Ni_1−x_(OH)_2_/CF: (A) Survey and (B) O 1s.


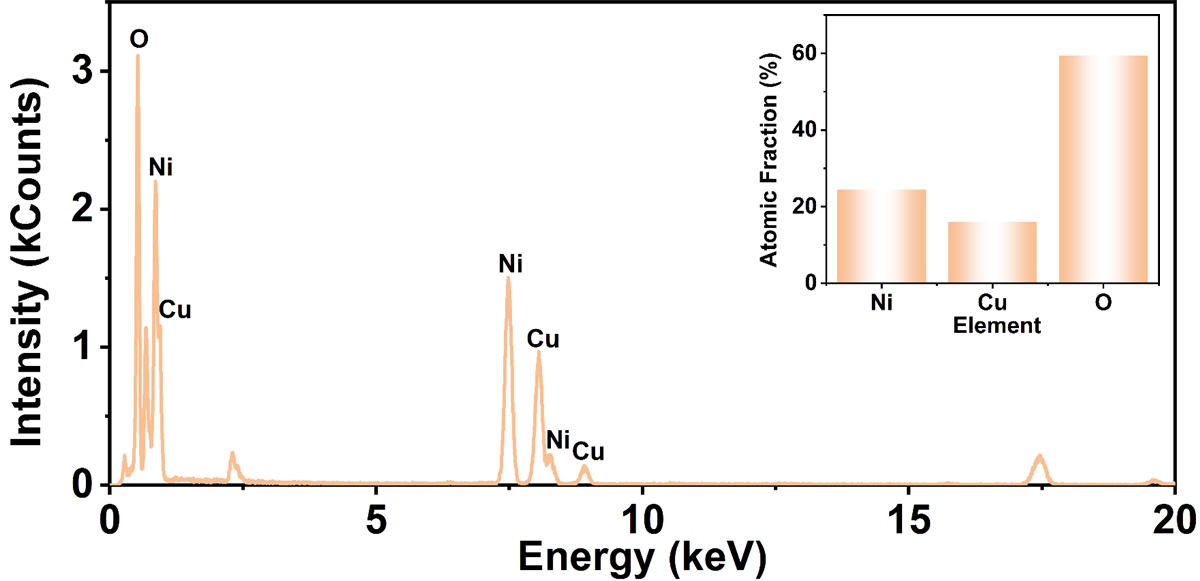


**FIGURE S12.** The elemental EDX results of Cu_x_Ni_1−x_(OH)_2_/CF.


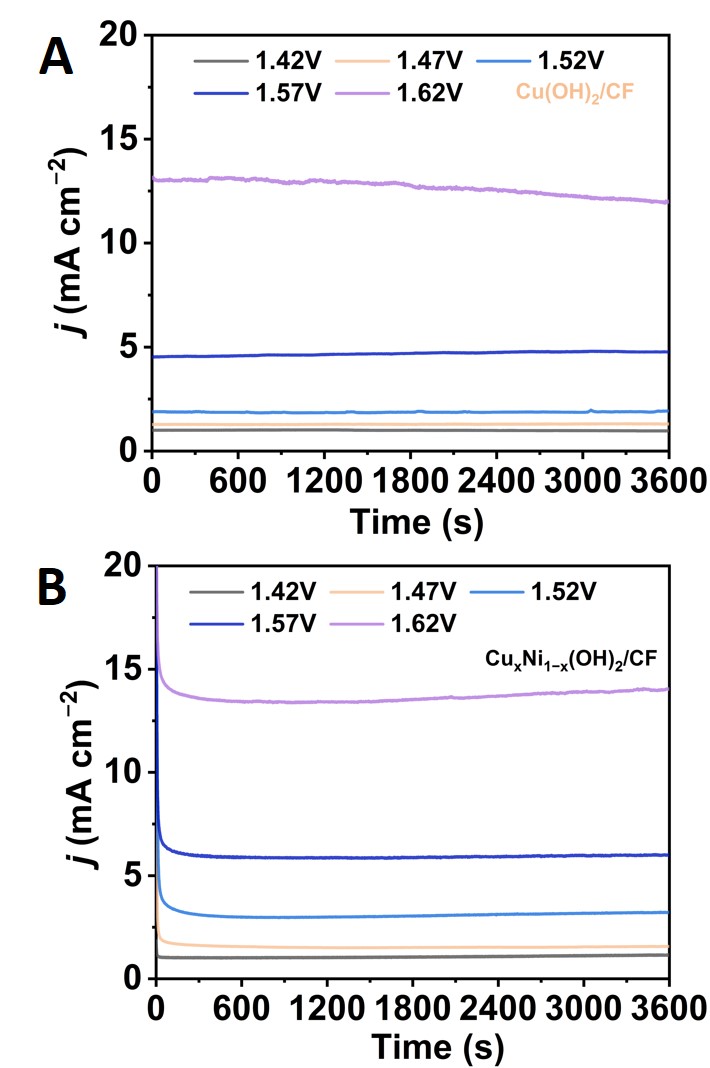


**FIGURE S13.** *I-t* curves for (A) Cu(OH)_2_/CF and (B) Cu_x_Ni_1−x_(OH)_2_/CF.


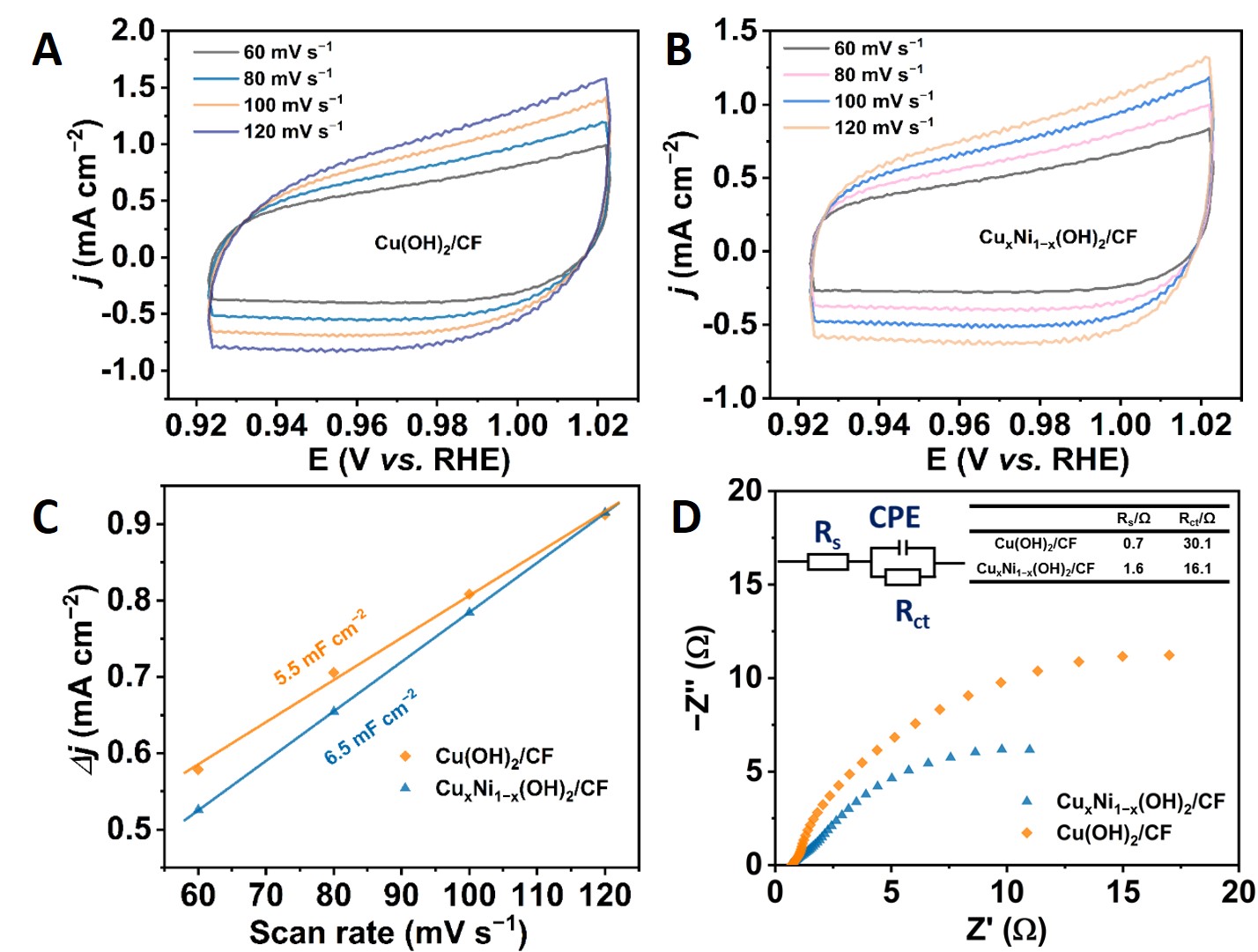


**FIGURE S14.** CV curves of (A) Cu(OH)_2_/CF and (B) Cu_x_Ni_1−x_(OH)_2_/CF at different scan rates; (C) capacitances inferred from charge current density diﬀerences against scan rates; (D) EIS plots.


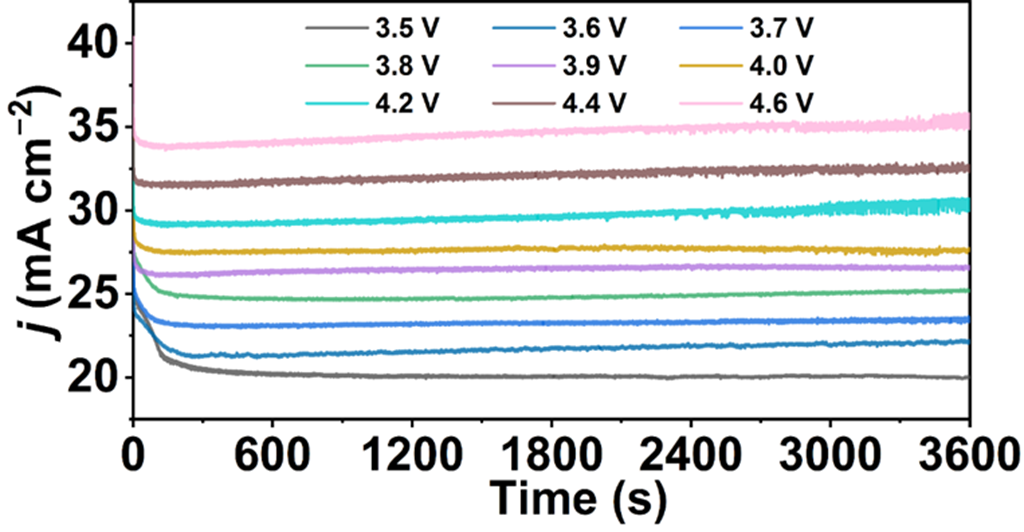


**FIGURE S15.** *I-t* curves of CO_2_RR//CHOR under different potentials.


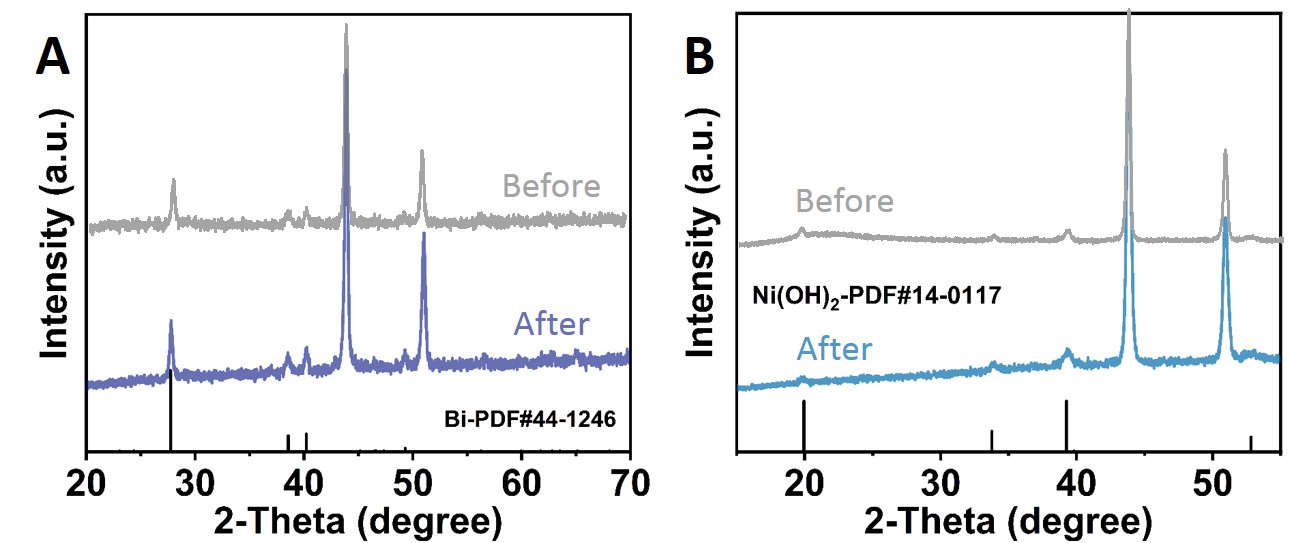


**FIGURE S16.** XRD patterns of (A) eBiCu/CF and (B) Cu_x_Ni_1−x_(OH)_2_/CF after coupling electrolysis.

**
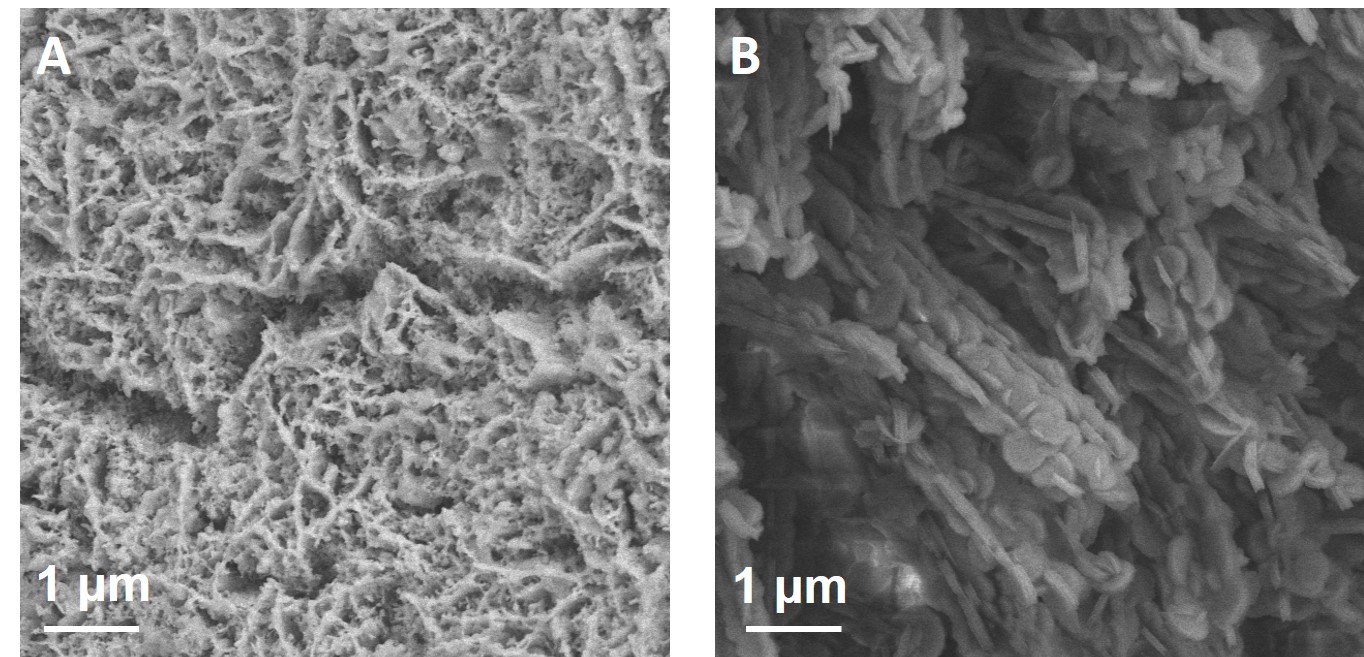
**

**FIGURE S17.** SEM images of (A) eBiCu/CF and (B) Cu_x_Ni_1−x_(OH)_2_/CF after coupling electrolysis.

**TABLE S1** CO_2_RR catalytic performances of recently reported Bi-based catalysts for formate production in the H-type cell.

| Catalyst | Electrolyte | Potential  (V vs. RHE) | FE (%) | Stability | Ref. |
| --- | --- | --- | --- | --- | --- |
| eBiCu/CF | 0.5 M KHCO_3_ | −0.78 ~ −1.18 | >90 | 30 h (~50 mA) | This work |
| Bimetallic Cu-Bi | 0.5 M KHCO_3_ | −0.90 ~ −1.10 | >90 | 20 h (~50 mA) | S1 |
| Bi-TiO_2_-700 | 0.1 M KHCO_3_ | −0.60 ~ −1.20 | >90 | 70 h (~8 mA) | S2 |
| Bi /Cu | 0.1 M KHCO_3_ | −1.46 | 95 | － | S3 |
| pits-Bi | 0.1 M KHCO_3_ | −1.14 | 95 | 10 h (~18 mA) | S4 |
| mpBi | 0.5 M NaHCO_3_ | -1.00 | 87 | 12 h (~5 mA) | S5 |
| NTD-Bi | 0.5 M KHCO_3_ | -1.05 | 90 | 48 h (~36 mA) | S6 |
| Bi NSs | 0.1 M KHCO_3_ | -0.85 | 85 | 8 h (~6 mA) | S7 |

**TABLE S2** CHOR catalytic performances of recent years.

| Catalyst | Electrolyte | Potential  (V vs. RHE) | FE (%) | Stability | Ref. |
| --- | --- | --- | --- | --- | --- |
| Cu_x_Ni_1−x_(OH)_2_/CF | 1 M NaOH + 0.1 M cyclohexanone | 1.52 V | >93 | 50 h | This work |
| Ni(OH)_2_-SDS | 0.5 M KOH +  20 mM cyclohexanone | 1.50 V | 93 | 20 h | S8 |
| Cu-Ni(OH)_2_ | 1 M NaOH + 0.1 M cyclohexanone | 1.62 V | ~90 | － | S9 |

References

[S1] Z. Li, B. Sun, D. Xiao, Z. Wang, Y. Liu, Z. Zheng, P. Wang, Y. Dai, H. Cheng, B. Huang, *Angew. Chem. Int. Ed.* **2023**, *62*, e202217569.

[S2] G. Jia, Y. Wang, M. Sun, H. Zhang, L. Li, Y. Shi, L. Zhang, X. Cui, T. W. B. Lo, B. Huang, J. C. Yu, *J. Am. Chem. Soc.* **2023**, *145*, 14133.

[S3] W. Lv, J. Zhou, J. Bei, R. Zhang, L. Wang, Q. Xu, W. Wang, *Appl. Surf. Sci.* **2017**, *393*, 191.

[S4] Y. Yuan, Q. Wang, Y. Qiao, X. Chen, Z. Yang, W. Lai, T. Chen, G. Zhang, H. Duan, M. Liu, H. Huang, *Adv. Energy Mater.* **2022**, *12*, 2200970.

[S5] E. Bertin, S. Garbarino, C. Roy, S. Kazemi, D. Guay, *J. CO_2_ Util.* **2017**, *19*, 276.

[S6] Q. Gong, P. Ding, M. Xu, X. Zhu, M. Wang, J. Deng, Q. Ma, N. Han, Y. Zhu, J. Lu, Z. Feng, Y. Li, W. Zhou, Y. Li, *Nat. Commun.* **2019**, *10*, 2807.

[S7] T. Gao, X. Wen, T. Xie, N. Han, K. Sun, L. Han, H. Wang, Y. Zhang, Y. Kuang, X. Sun, *Electrochim. Acta* **2019**, *305*, 388.

[S8] Z. Li, X. Li, H. Zhou, Y. Xu, S.-M. Xu, Y. Ren, Y. Yan, J. Yang, K. Ji, L. Li, M. Xu, M. Shao, X. Kong, X. Sun, H. Duan, *Nat. Commun.* **2022**, *13*, 5009.

[S9] R. Wang, Y. Kang, J. Wu, T. Jiang, Y. Wang, L. Gu, Y. Li, X. Yang, Z. Liu, M. Gong, *Angew. Chem. Int. Ed.* **2022**, *61*, e202214977.
